# Impact of an intervention for perinatal anxiety on breastfeeding: findings from the *Happy Mother—Healthy Baby* randomized controlled trial in Pakistan

**DOI:** 10.1186/s13006-024-00655-8

**Published:** 2024-08-02

**Authors:** Anum Nisar, Haoxue Xiang, Jamie Perin, Abid Malik, Ahmed Zaidi, Najia Atif, Atif Rahman, Pamela J. Surkan

**Affiliations:** 1 Institute of Population Health, University of Liverpool, Liverpool, UK; 2 Human Development Research Foundation, Rawalpindi, Pakistan; 3 Bloomberg School of Public Health, Johns Hopkins University, Baltimore, Maryland USA; 4 Health Services Academy, Islamabad, Pakistan

**Keywords:** Breastfeeding, Prenatal anxiety, Cognitive-behavioural therapy, Intervention, Pakistan

## Abstract

**Background:**

The study examined the effects of Happy Mother—Healthy Baby (HMHB), a cognitive-behavioural therapy (CBT) intervention on breastfeeding outcomes for Pakistani women with prenatal anxiety.

**Methods:**

Breastfeeding practices were evaluated in a randomized controlled trial between 2019 and 2022 in a public hospital in Pakistan. The intervention group was randomized to receive six HMHB sessions targeted towards prenatal anxiety (with breastfeeding discussed in the final session), while both groups also received enhanced usual care. Breastfeeding was defined in four categories: early breastfeeding, exclusive early breastfeeding, recent breastfeeding, and exclusive recent breastfeeding. Early breastfeeding referred to the first 24 h after birth and recent breastfeeding referred to the last 24 h before an assessment at six-weeks postpartum. Potential confounders included were mother’s age, baseline depression and anxiety levels, stress, social support, if the first pregnancy (or not) and history of stillbirth or miscarriage as well as child’s gestational age, gender. Both intent-to-treat and per-protocol analyses were examined. Stratified analyses were also used to compare intervention efficacy for those with mild vs severe anxiety.

**Results:**

Out of the 1307 eligible women invited to participate, 107 declined to participate and 480 were lost to follow-up, resulting in 720 women who completed the postpartum assessment. Both intervention and control arms were similar on demographic characteristics (e.g. sex, age, income, family structure). In the primary intent-to-treat analysis, there was a marginal impact of the intervention on early breastfeeding (OR 1.38, 95% CI: 0.99–1.92; 75.4% (*N* = 273) vs. 69.0% (*N* = 247)) and a non-significant association with other breastfeeding outcomes (OR1.42, 95% CI: 0.89–2.27; (47) 12.9% vs. (34) 9.5%, exclusive early breastfeeding; OR 1.48, 95% CI: 0.94–2.35; 90% (*N* = 327) vs. 86% (*N* = 309), recent breastfeeding; OR1.01, 95% CI: 0.76–1.35; 49% (*N* = 178) vs 49% (*N* = 175) exclusive recent breastfeeding). Among those who completed the intervention’s six core sessions, the intervention increased the odds of early breastfeeding (OR1.69, 95% CI:1.12–2.54; 79% (*N* = 154) vs. 69% (*N* = 247)) and recent breastfeeding (OR 2.05, 95% CI:1.10–3.81; 93% (*N* = 181) vs. 86% (*N* = 309)). For women with mild anxiety at enrolment, the intervention increased the odds of recent breastfeeding (OR 2.41, 95% CI:1.17–5.00; 92% (*N* = 137) vs. 83% (*N* = 123).

**Conclusions:**

The study highlights the potential of CBT-based interventions like HMHB to enhance breastfeeding among women with mild perinatal anxiety, contingent upon full participation in the intervention.

**Trial registration:**

ClinicalTrials.gov NCT03880032.

**Supplementary Information:**

The online version contains supplementary material available at 10.1186/s13006-024-00655-8.

## Background

Lactation has developed through evolutionary processes to create an optimal system for delivering essential nutrients in sufficient quantities from mothers to their offspring [1]. Breastfeeding has considerable impacts on children's cognition, behavior, physical growth and development, as well as effects on the mothers’ physical and psychological wellbeing [2, 3, 4]. The World Health Organization (WHO) suggests that breastfeeding should continue exclusively, meaning that the infant is only fed with breastmilk, for at least six months [5]. Exclusive breastfeeding can lead to a 10% reduction in the disease burden among children below the age of five [6]. Pakistan, with more than five million children born each year, has one of the highest numbers of births in the world [7]. However, exclusive breastfeeding practices in Pakistan have fallen short of recommended targets. According to the Pakistan Demographic and Health Survey, only 48% of children less than six months of age are exclusively breastfed, while 53% of children receive any breastmilk until the age of two years old [8]. This means only approximately half of children under 6 months are exclusively breastfed, indicating a need for improved breastfeeding practices in Pakistan.

Perinatal anxiety can negatively affect maternal functioning, resulting in emotional distress, and potential disruptions in the formation of the mother-infant bond as well as less likelihood of breastfeeding [9, 10, 11]. Low breastfeeding self-efficacy is a major contributor to discontinuation of exclusive breastfeeding [12]. Cognitive behavioural therapy (CBT) has been recommended to promote breastfeeding in pregnant and new mothers in lower- and middle-income countries (LMICs) by promoting counselling, social support, education and women’s empowerment [13, 14].

Given that women with anxiety are at a higher risk of discontinuing exclusive and continued breastfeeding practices [9, 10, 15], we sought to evaluate a CBT-based intervention, called *Happy Mother, Healthy Baby* (HMHB), for women with prenatal anxiety in Pakistan that included a session involving the discussion of and encouraged support for breastfeeding. In follow-up assessment at six weeks after birth, HMHB was effective in reducing the odds of depression by 81% (OR 0.19, 95% CI: 0.13–0.28), with 11.6% (*N* = 44) of intervention participants with postpartum depression versus 40.5% (*N* = 152) of control participants with postpartum depression. HMHB also reduced the odds of moderate to severe symptoms of anxiety by 74% (OR 0.26, 95% CI: 0.17–0.40), with 8.7% (*N* = 33) of intervention participants versus 26.7% (*N* = 100) control participants having moderate-to-severe postpartum anxiety symptoms [16]. Given the inclusion of guidance on breastfeeding in the intervention, we sought to evaluate the effect of an anxiety-focused maternal mental health intervention using CBT on breastfeeding outcomes among women with symptoms of at least mild anxiety in Pakistan.

## Methods

### Study setting and participant recruitment

Data for this study were obtained from a single-blinded randomized controlled trial to study the effectiveness of the Healthy Mother—Happy Baby (HMHB) intervention to reduce anxiety among pregnant women (clinicaltrial.gov identifier: NCT03880032) [17]. The study recruited 1200 women from Holy Family Hospital (HFH), a public facility in Rawalpindi, Pakistan, between 16th April 2019 until 31 January 2022. HFH is located in Rawalpindi, Pakistan, is a large facility with around 900 beds, making it a major regional healthcare facility. Annually, around 5,000 births are delivered at HFH, evidence of its crucial role in maternal and neonatal care in the region where it serves a diverse population from urban, rural, and semi-urban areas. All participants were recruited by female research assistants in the outpatient Gynaecology and Obstetrics Department during their initial prenatal visit. Participants were followed up at six-weeks after birth.

### Screening and inclusion criteria

The study employed three levels of inclusion/exclusion screening criteria during the enrolment process. In the first level, women had to be at ≤ 22 weeks' gestation, ≥ 18 years old, reside ≤ 20 km from HFH, and have a basic understanding of Urdu. Women who met these criteria and showed willingness to participate were asked to provide informed consent. At the second level of screening, potential participants were excluded if reporting life-threatening health conditions, such as active severe depression or suicidal ideation. Other exclusions included self-reported significant learning disabilities, a self-reported psychiatric disorder or ongoing psychiatric care, medical disorders or severe maternal morbidity requiring inpatient management, and ICU admission indicated by diagnosis (not solely for assessment purposes), past or current significant learning disabilities, past or current psychiatric disorders, medical disorders, or severe maternal morbidity. At the third level of screening, potential participants were assessed for the presence of at least mild anxiety using the Hospital Anxiety Depression Scale (HADS) screening tool. Those who scored ≥ 8 on the HADS anxiety sub-scale (indicating at least mild anxiety) were interviewed by trained assessors who conducted a Structured Clinical Interview for DSM IV Diagnoses (SCID) to rule out depression. Women who met the conditions for a major depressive episode (MDE) were not included. MDE was defined using a diagnostic semi-structured guide from the Structured Clinical Interview for the Diagnostic and Statistical Manual (SCID), which is based on American Psychiatric Association's Diagnostic and Statistical Manual for Mental Disorders (DSM). Assessment with this method is considered equivalent to a clinical diagnosis in line with the DSM criteria.

### Randomization

Study participants were randomly assigned to either the intervention or control group using a pseudo random-number generator. The random sequence was assigned through permuted blocks of size 4, 8, 12, and 16. The assignment list was printed in order, with each assignment separately stored in opaque envelopes and numbered sequentially. Once an eligible individual consented to participate in the study, the research team proceeded by selecting the next available envelope to determine the individual's assignment into either the intervention or control arm. Throughout this process, the trial team, comprising the assessment team, principal investigators, and co-investigators, remained blinded to the allocation.

### Study groups

Those randomized to the intervention group received the HMHB program, an intervention relying on principles of Cognitive Behavioural Therapy (CBT) that aimed to reduce symptoms of anxiety during pregnancy [18]. It was adapted from WHO endorsed psychosocial intervention for perinatal depression called the Thinking Healthy Programme [19]. The intervention was delivered by non-specialist providers (with a two-year bachelor’s degree and a two-year master’s degree in psychology but no clinical experience). They were trained on the intervention and received regular weekly group supervision over the trial period. HMHB was designed to target risk factors for anxiety that women experienced during pregnancy that were identified in our preliminary research [18]. It also integrated stress management techniques, such as breathing exercises. To make the intervention more culturally appropriate, personalized illustrations were used in order to facilitate guided discovery, behavioural activation, stress reduction, and convey essential health messages [18]. The sessions were supplemented by take-home exercises.

HMHB consisted of six core weekly sessions and up to six optional booster sessions (delivered as needed). The first five weekly one-on-one sessions were intended for early to mid-pregnancy. The final sixth core session was given in the third trimester of pregnancy. This session was aimed to help manage anxiety during late pregnancy, prepare for baby’s arrival, and navigate the early post-natal period. It highlighted the importance of breastfeeding and providing colostrum as a pre-lacteal feed instead of culturally common practices involving feeding infants honey or herbal tonics. It also encouraged family support for mothers to breastfeed.

The control group received enhanced usual care at the Gynaecology and Obstetrics Department. Usual care recommended at the study hospital typically involves up to eight visits for evaluating health status, discussing any concerns, and performing routine exams consistent with the stage of pregnancy. The care of women participating in HMHB was enhanced by reminders for study visits, expedited care (shorter wait times), as well as reimbursement for transportation to visits and for as many ultrasounds as were medically indicated at HFH during pregnancy.

### Breastfeeding indicators

In line with WHO definition of exclusive breastfeeding, women who confirmed providing only colostrum/breastmilk within the first 24 h and reported no use of formula, Ghutti (traditional pre-lacteal feed), herbal water, tea, or other animal milk were considered to have engaged in ‘early exclusive breastfeeding’. Breastfeeding women who reported both breastmilk and at least one other nutritional source fell into the ‘early breastfeeding’ category. We also assessed breastfeeding at six weeks postpartum by asking mothers if they were breastfeeding and if they had given breast milk or any other nutrition to their infants to determine whether it was exclusive or non-exclusive. These women were categorized as having engaged in ‘recent exclusive breastfeeding’ if no other nutrition source was provided or ‘recent breastfeeding’ if receiving both breastmilk and other nutrients. Our indicators for 'recent' breastfeeding practices, namely 'recent exclusive breastfeeding' and 'recent breastfeeding', pertain to exclusive and non-exclusive breastfeeding within 24 h before the six-week postpartum assessment. We chose the term 'recent' because it covers both exclusively breastfed infants and those receiving some breastmilk, unlike the WHO definition of 'predominant breastfeeding', which focuses solely on the infant's main source of nourishment.

### Covariates

#### The hospital anxiety and depression scale

The Hospital Anxiety and Depression Scale (HADS) is a well-known instrument that includes 14 items rated on a 4-point scale that has been validated in numerous languages and settings [20, 21]. The HADS focuses on non-physical symptoms to screen for anxiety and depression, but does not include all of the diagnostic criteria of depression specified in the Diagnostic and Statistical Manual of Mental Disorders (DSM) [21]. For example, it does not include items on appetite, sleep and self-harm/suicidal thoughts [21]. It comprises two distinct subscales to assess anxiety and depression, each containing 7 items with scores ranging from 0 to 21. Typical symptom cut-offs are 0–7 (normal), 8–10 (mild), 11–14 (moderate), and 15–21 (severe). A cut-off of ≥ 8 was defined as the threshold for being ‘at risk’ for anxiety or depression. The Urdu adaptation of this scale has been previously modified for use in Pakistan and has been administered successfully [22] including in pregnant women [23], showing satisfactory reliability, validity, and high concurrence with the English version for use of the symptom threshold of ≥ 8 [24] when assessing antenatal anxiety and depression in Pakistan [25].

#### Perceived stress scale (PSS-10)

The PSS-10 is a validated global measure of perceived stress that consists of 10 items scored on a 5-point scale ranging from 0 to 4 (maximum score 40) [26]; higher scores indicate more stress. It has been adapted for use in Pakistan [27]. A score of ≥ 20 corresponds to high stress.

#### Multi-dimensional scale of social support (MSPSS)

The Multi-dimensional Scale of Perceived Social Support (MSPSS) is a 12-item measure of subjective availability of support (primarily emotional) which has been validated and successfully adapted to the Pakistani context [28]. Scores are on a 7-point scale (1 = very strongly disagree; 7 = very strongly agree), with higher scores indicating more support.

### Statistical analyses

Descriptive statistics were performed to investigate postpartum symptoms of depression and anxiety in relation to breastfeeding. In women with completed measures of breastfeeding outcomes, we examined the differences at baseline between intervention and control arms to verify that randomization generated comparable arms. We used standard statistical comparisons, including Chi-square test for categorical factors and Student’s t-test for continuous factors, to determine the statistical significance of any differences between arms. All analyses followed the intent-to-treat (ITT) principle unless otherwise noted, comparing the four breastfeeding outcomes in the groups to which they were randomized.

We compared early breastfeeding, exclusive early breastfeeding, recent breastfeeding, and exclusive recent breastfeeding outcomes between arms, where ‘early’ was defined as first 24 h after childbirth and ‘recent’ was defined as within the last 24 h before the assessment that occurred approximately six weeks postpartum. Comparisons between arms following the principle of ITT were estimated with logistic regression. In addition to the intent-to-treat analysis among all participants, we also performed a stratified analysis to separately estimate the intervention effects for women who had mild anxiety levels (HADS anxiety score: 8–11) and the intervention effects for women who had moderate to severe anxiety levels (HADS anxiety score: 11–21) at enrolment.

We also conducted an analysis to examine breastfeeding outcomes in a subset of women randomized to the intervention arm who only included those that received all six intervention sessions (“intervention completers”). We adjusted for potential confounders including gestational age, depression and anxiety at enrolment, stress at enrolment measured with the Perceived Stress Scale (PSS-10), general social support and social support from family (both measured with the Multidimensional Scale of Social Support (MSPSS)), maternal age, child’s sex, whether first pregnancy or not and history of stillbirth or miscarriage. Selection of confounding factors for adjustment was based on prior knowledge of what was expected to influence intervention session receipt and by examining the baseline variables for those with six intervention sessions compared to those in the control arm. Given breastfeeding was a secondary outcome, this study was not specifically powered to detect differences related to breastfeeding outcomes. Rather, it was powered to detect a difference in the six-week postpartum mental health outcomes of participants between arms among 1,200 enrolled participants with a 30% expected dropout rate.

Finally, we evaluated a dose response relationship for the intervention considering the receipt of booster sessions. Using the Cochrane Armitage test for trend, we examined this relationship for the four types of breastfeeding considered, across three dose groups, 1) control (no intervention), 2) only core intervention sessions, and 3) core and booster sessions.

### Ethics

Ethical approval for this research was received from the Institutional Review Boards of the Johns Hopkins Bloomberg School of Health (IRB No. 00009177; Approved April 2, 2019), the Human Development Research Foundation Ethics Committee (IRB/001/2017; Approval March 10, 2017), and Global Mental Health Data Safety and Monitoring Board appointed by the National Institute of Mental Health (NIMH) in the United States (No assigned approval number; Approved March 11, 2019). Prior to their involvement in this study, all participants provided written informed consent, indicating their willingness to take part in the research.

## Results

Out of over 91 thousand women screened, 1307 women met the inclusion criteria, including having moderate to high anxiety symptoms and not meeting the diagnostic criteria for clinical depression. Of these 1,307, 1,200 (92%) agreed to participate and were enrolled in the trial. Among the 1200 pregnant women who were enrolled 445 (37%) were lost to follow-up, mostly because they were unreachable or because they declined to participate later. The remaining 755 (63%) of enrolled pregnancies were followed until six weeks postpartum. Of those, 720 had complete measures on breastfeeding outcomes.

**Fig. 1:**
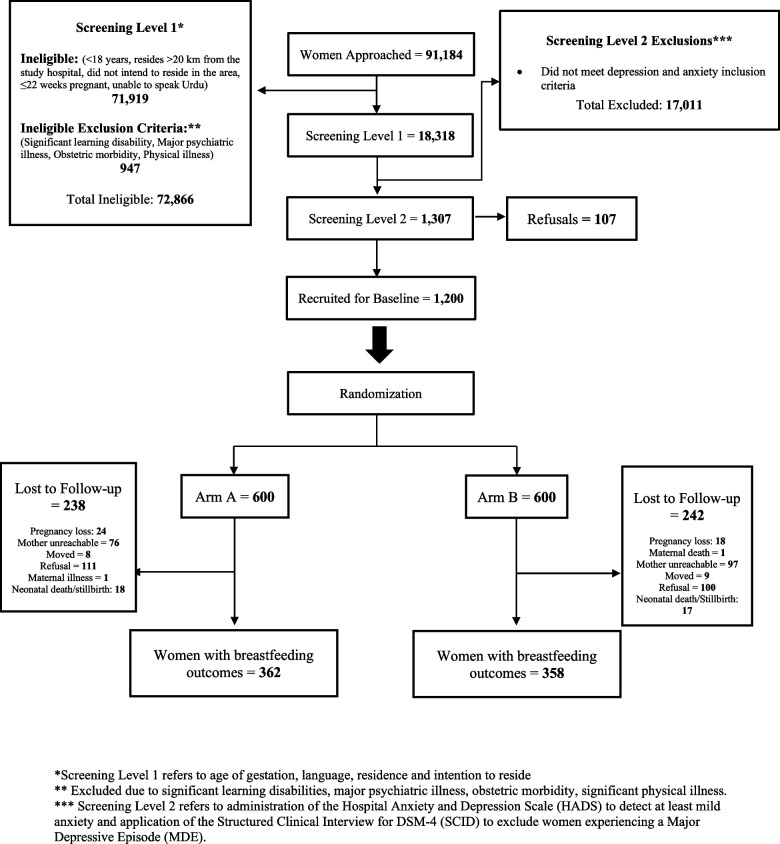
Consort diagram

Descriptive statistics showed that breastfeeding was inversely related to symptoms of both depression and anxiety (Supplementary Table 1). The women were similar between arms at enrolment across all characteristics examined. This included maternal age (mean (SD) 25.1 (4.7) vs 25.3 (4.5) for intervention and control arms respectively; *p* = 0.519), whether it was the participant’s first pregnancy (98 (27%) vs 109 (30%); *p* = 0.359), having at least one other child at time of enrolment in pregnancy (204 (56%) vs 207 (58%); *p* = 0.104), and the participant having a history of stillbirth or miscarriage (160 (44%) vs 143 (40%), *p* = 0.280). Education, family structure, social support, and self-reported monthly income were also similar between arms. Participant baseline anxiety symptoms were mean (SD) 11.2 (2.0) vs 11.2 (1.9), depression 6.9 (2.9) vs 6.6 (2.6), in the intervention and control arms respectively. Perceived stress was also examined for differences and was similar across arms. A detailed description of participants with known breastfeeding outcomes by arm is shown in Table 1 and Fig. 1.

**Table 1:** Description of the 720 women with measured breastfeeding at six-weeks postpartum enrolled in the HMHB trial to be used in the intent-to-treat analysis

	**Overall** **(*N* = 720)**	**Intervention Arm** **(*N* = 362)**	**Control Arm** **(*N* = 358)**
	Mean (SD)	Mean (SD)	Mean (SD)
Age (years)	25.2 (4.6)	25.1 (4.7)	25.3 (4.5)
Gestational Age (weeks)	38.1 (2.0)	38.1 (2.1)	38.1 (1.9)
Stress at enrollment (PSS-10)	1.00 (1.35)	1.04 (1.40)	0.97 (1.30)
Anxiety at enrollment (HADS)	11.2 (1.9)	11.2 (2.0)	11.2 (1.9)
Depression at enrollment (HADS)	6.7 (2.8)	6.9 (2.9)	6.6 (2.6)
Major social support (MSPSS)	3.5 (0.9)	3.5 (1.0)	3.6 (0.9)
Social support from family (MSPSS)	3.5 (1.0)	3.4 (1.1)	3.5 (0.9)
Social support from friend (MSPSS)	2.7 (1.3)	2.7 (1.3)	2.7 (1.3)
Maternal age ≤ 25	432 (60%)	212 (59%)	220 (61%)
Child’s gender (male)	352 (49%)	178 (49%)	174 (49%)
First pregnancy (yes)	207 (29%)	98 (27%)	109 (30%)
≥ 1 child from a prior pregnancy	411 (57%)	204 (56%)	207 (58%)
History of stillbirth or miscarriage (yes)	303 (42%)	160 (44%)	143 (40%)
Education level			
≤ Primary school	177 (24%)	92 (25%)	85 (24%)
Middle school – matriculation	336 (47%)	167 (46%)	169 (47%)
≥ Intermediate	207 (29%)	103 (28%)	104 (29%)
Family structure			
Nuclear	231 (32%)	119 (33%)	112 (31%)
Joint (parents)	245 (34%)	120 (33%)	125 (35%)
Extended (parents and siblings)	206 (29%)	99 (27%)	107 (30%)
Monthly income (PKR^a^ (USD^b^)			
Low (< 20,000 (< 100 USD)	335 (47%)	163 (45%)	172 (48%)
Middle (20,000 – 35,000 (100–1256 USD)	278 (39%)	140 (39%)	138 (39%)
High (> 35,000 (> 125 USD)	88 (12%)	47 (13%)	41 (11%)
Anxiety at enrollment (HADS)			
Mild (≥ 8 to ≤ 10)	298 (41%)	149 (41%)	149 (42%)
Moderate (≥ 11 to ≤ 15)	403 (56%)	205 (57%)	198 (55%)
Severe (≥ 16 to ≤ 21)	19 (3%)	8 (2.2%)	11 (3%)

Social support is defined as the provision of emotional, informational, appraisal, and instrumental from others in one’s social network. For family structure, nuclear family structure refers to the participant, her husband and children; joint family structure refers to a participant living with her husband, children and in-laws; extended family structure includes living with not only in-law parents but also sister- and brothers-in-laws and potentially their families. ^a^Pakistani Rupee

^b^United States Dollar

We estimated the intervention effects on the four measured breastfeeding outcomes, including early breastfeeding, exclusive early breastfeeding, recent breastfeeding, and exclusive recent breastfeeding, by comparing results of 362 women in the intervention arm and 358 women in the control arm. Overall, in the ITT analysis did not show statistical differences (*p*-value > 0.05) for any of the four breastfeeding outcomes between the intervention and control arms, although there was marginal evidence of an intervention effect on early breastfeeding (75.4% vs. 69.0% with *p*-value = 0.06). The detailed results for all four breastfeeding outcomes are shown in Table 2.

**Table 2:** Estimated intervention effects (intervention arm relative to control arm) among 720 women with measured breastfeeding results in the HMHB trial

			**Unadjusted Odds Ratio**	**Adjusted Odds Ratio**
	**Intervention Arm (*n* = 362)** **N (%)**	**Control Arm** **(*n* = 359)** **N (%)**	**Estimate (95% CI)**^a^	**Estimate (95% CI)**^b^
Early breastfeeding	273 (75.4%)	247 (68.8%)	1.38 (0.99, 1.92)	1.38 (0.98, 1.92)
Exclusive early breastfeeding	47 (12.9%)	34 (9.5%)	1.42 (0.89, 2.27)	1.44 (0.90, 2.32)
Recent breastfeeding	327 (90.3%)	309 (86.1%)	1.48 (0.94, 2.35)	1.49 (0.93, 2.40)
Exclusive recent breastfeeding	178 (49.2%)	175 (48.7%)	1.01 (0.76, 1.35)	0.99 (0.74, 1.34)

Early breastfeeding refers to the first 24 h after birth. Recent breastfeeding refers to the last 24 h before the assessment at six-weeks postpartum

^a^95% Confidence Intervals (CI) determined using logistic regression

^b^Adjusted for gestational age, depression at enrolment (HADS), stress at enrolment (PSS-10), major social support (MSPSS), social support from family (MSPSS), maternal age, child’s sex, whether first pregnancy or not and history of stillbirth or miscarriage using logistic regression

We also performed an exploratory analysis to compare intervention effects stratifying by anxiety symptoms levels at enrolment (Table 3). The estimated intervention impact among women with mild anxiety at enrolment was somewhat larger than among women with high baseline anxiety levels. We found that for women with mild anxiety, the intervention increased the odds of recent breastfeeding (92% vs. 83%, odds ratio (OR) 2.41, 95% CI: 1.17 to 5.00). A summary comparison by arm among women with mild anxiety and women with moderate to severe anxiety is included in Supplementary Tables 2 and 3, respectively.

**Table 3:** Comparison of estimated intervention effects between arms among 298 women who had a mild level of anxiety and 422 women who had moderate- to severe- anxiety levels (HADS)^a^ at enrolment with measured breastfeeding in the HMHB trial

			*Unadjusted Odds ratio*^b^* (reference: control group)*	*Adjusted Odds ratio*^c^* (reference: control group)*
	**Intervention Arm** **N (%)**	**Control** **Arm** **N (%)**	**Estimate (95% CI)**	**Estimate (95% CI)**
**Women with mild anxiety levels*** (N* = *298)*				
Early breastfeeding	111 (74%)	104 (70%)	1.26 (0.76, 2.11)	1.23 (0.72, 2.10)
Exclusive early breastfeeding	14 (9%)	19 (13%)	0.71 (0.34, 1.47)	0.69 (0.33, 1.46)
Recent breastfeeding	137 (92%)	123 (83%)	2.41 (1.17, 5.00)	2.46 (1.16, 5.23)
Exclusive recent breastfeeding	80 (54%)	66 (44%)	1.46 (0.92, 2.30)	1.49 (0.93, 2.39)
**Women with moderate to severe anxiety levels*** (N* = *422)*				
Early breastfeeding	162 (76%)	143 (68%)	1.47 (0.96, 2.26)	1.39 (0.90, 2.16)
Exclusive early breastfeeding	33 (15%)	15 (7%)	2.37 (1.25, 4.51)	2.48 (1.29, 4.78)
Recent breastfeeding	190 (89%)	186 (89%)	1.02 (0.55, 1.89)	0.97 (0.51, 1.84)
Exclusive recent breastfeeding	98 (46%)	109 (52%)	0.78 (0.53, 1.15)	0.76 (0.51, 1.13)

^a^Mild anxiety level: (HADS: 8–10), moderate to severe anxiety level: (HADS: 11–21)

^b^Estimate by logistic regression

^c^Adjusted for gestational age, depression at enrolment (HADS), stress at enrolment (PSS-10), major social support (MSPSS), social support from family (MSPSS), maternal age, child’s sex, whether first pregnancy or not and history of stillbirth or miscarriage using logistic regression

In addition to our primary analysis, which was completed following the principle of intent-to-treat, we conducted additional analysis only involving 195 women in the intervention arm who received six intervention sessions (“intervention completers”), compared with all 358 women in control arm. As shown in Table 4, for participants receiving six intervention sessions, the intervention increased the odds of early breastfeeding (79% vs. 69%, OR 1.69, 95% CI: 1.12 to 2.54) and recent breastfeeding (93% vs. 86%, OR 2.05, 95% CI: 1.10 to 3.81). Women receiving six intervention sessions, although not randomized to receive the complete intervention, were similar to those in the control arm (Supplementary Table 4).

**Table 4:** Estimated intervention effects (intervention arm relative to control arm) among 358 women in the control arm, and 195 women in the intervention arm receiving six intervention sessions in the HMHB trial

	**Intervention Arm**	**Control** **Arm**	**Unadjusted Odds Ratio**^a^	**Adjusted Odds Ratio**^b^
	**N (%)**	**N (%)**	**Estimate (95% CI)**	**Estimate (95% CI)**
Early breastfeeding	154 (79%)	247 (69%)	1.69 (1.12, 2.54)	1.62 (1.06, 2.47)
Exclusive early breastfeeding	24 (12%)	34 (9%)	1.34 (0.77, 2.32)	1.27 (0.72, 2.24)
Recent breastfeeding	181 (93%)	309 (86%)	2.05 (1.10, 3.81)	1.93 (1.02, 3.65)
Exclusive recent breastfeeding	102 (52%)	175 (49%)	1.15 (0.81, 1.63)	1.14 (0.80, 1.63)

^a^Significance determined by logistic regression

^b^Adjusted for gestational age, depressive and anxiety symptoms at enrolment (HADS), stress at enrolment (PSS-10), major social support (MSPSS), social support from family (MSPSS), maternal age, child’s gender, whether first pregnancy or not and history of stillbirth or miscarriage using logistic regression

Finally, according to the test for trend to test differences between receiving no intervention, the six core sessions and six core and booster sessions, we found no significant association between any type of breastfeeding and dose of intervention (Supplemantary Table 6).

## Discussion

The overall findings of the intent-to-treat analysis (including women who dropped out and did not receive all sessions) demonstrated no significant differences in any of the breastfeeding outcomes between the intervention and control arms. However, our results suggest that the HMHB intervention promoted early breastfeeding initiation and continuation for women who received the full six core sessions of the program. It is important to note that the intervention content overall did not primarily target breastfeeding and that content related to women’s perinatal well-being (focused on anxiety reduction) and the discussion of breastfeeding was presented only during the last visit of the program.

In other words, our finding of a significant impact only for those receiving the complete intervention dose may be because the relevant content was in the final session. Specifically, we observed an increase in both the odds of early breastfeeding initiation and in the odds of women continuing breastfeeding among women who attended the full intervention, compared to women in the control arm. Another study from Kenya showed that a series of home-based breastfeeding counselling sessions proved more effective in promoting exclusive breastfeeding compared to a single facility-based session, which was deemed insufficient [29]. Women’s health programs that provide personalized support during the perinatal period have demonstrated success in enhancing mental health and promoting breastfeeding outcomes in varied settings and contexts [30]. Given social support was also a component of several intervention sessions, it could also have potentially played a role in promoting breastfeeding behaviours.

A recent systematic review of 76 studies with 79 comparisons of breastfeeding interventions from 30 low- and middle-income countries showed almost every intervention increased exclusive breastfeeding rates [31]. In a study of pregnant women from a rural district in the northwest province of Pakistan, Sikander et al., found that compared with routine counselling, counselling using principles of CBT not only significantly prolonged the duration of exclusive breastfeeding but also doubled its rates at six months postpartum [13]. Studies conducted in other resource-constrained settings in Syria [32], India [33], and Bangladesh [34] and sub-Saharan Africa [35] have also shown effectiveness of home-based interventions aiming to promote breastfeeding behaviours among the mothers, even when delivered by non-specialist providers. Table 5 shows a comparison of different psychosocial interventions and their effects on breastfeeding in context of different LMICs.

**Table 5:** Comparison of psychosocial interventions for breastfeeding in lower- and middle-income countries^a^

	**HMHB—Pakistan**	**Syria **[32]	**India** [33]	**Bangladesh** [34]	**Pakistan** [13]	**Uganda, Burkina Fasso, and South Africa** [35]	**Kenya** [29]
Intervention goal	To use cognitive behavioral therapy to reduce prenatal anxiety and to facilitate participants wellbeing, social support, and bonding with their baby during pregnancy	To provide medical follow up, educate provide emotional support, check on breastfeeding, check on maternal-child relationship, discuss problems and help women who have given birth, discuss family planning	To promote exclusive breastfeeding until 6 months of age (as well as assess effects on diarrhea and child growth)	To educate and counsel mothers about exclusive breastfeeding and early initiation of breastfeeding	To use cognitive-behavioral therapy to increase the rate and duration of exclusive breastfeeding in the first six months postpartum	To determine the effect of home-based breastfeeding counselling by peer counsellors	To determine the impact of facility-based semi-intensive and home-based intensive counselling in improving exclusive breast-feeding
Time point	Antenatal	Postnatal	Postnatal	Antenatal and postnatal	Antenatal and postnatal	Antenatal and postnatal	Antenatal and postnatal
Frequency of delivery	Weekly for 5 visits in early to mid-pregnancy, with a 6th visit in the 3rd trimester	At least once or in a series of 4 home visits	A series of 12 monthly visits until the child reached the age of one	15 sessions	7 sessions	5 sessions	7 sessions
Location	Health facility	Home	Home	Home	Home	Home	Home and health facility
Delivery agent	Non-specialized providers	Trained midwives	Community health workers and nutrition workers	Peer counsellors	Community health workers	Trained peers	Trained peers
Outcome(s) related to breastfeeding	Exclusive and recent breastfeeding both within 24 h of birth and within 24 h of an assessment at six weeks postpartum	Exclusive breastfeeding and breastfeeding practices (see below for examples) at 4 months postpartum	Primary: Exclusive breastfeeding at 3 months postpartum; Secondary: Exclusive breastfeeding at 4, 5, and 6 months of life	Prevalence of exclusive breastfeeding at 5 months postpartum and timing of initiation of breastfeeding	Rate and duration of exclusive breastfeeding at 6 months postpartum	Prevalence of exclusive breastfeeding at 12 and 24 weeks	Prevalence of exclusive breastfeeding at 6 months
Result(s) related to breastfeeding	HMHB had a marginally significant impact on early breastfeeding i.e. in the first 24 h of life (75.4% HMHB vs. 69.0% controls; OR 1.4, 95% CI: 0.99–1.92). In unadjusted per protocol analyses, HMHB increased the odds of early (OR 1.7, 95% CI:1.2–2.6) and recent breastfeeding i.e. measured in the prior 24 h at six-weeks after birth (OR 2.1, 95% CI:1.1–4.0)	A significantly higher proportion of mothers who received four doses or one dose of the intervention, respectively, exclusively breastfed their infants (28.5% and 30%, respectively) compared to controls who received no intervention (20%), *p* = 0.02. However, no differences were found between these groups for other breastfeeding outcomes, e.g. breastfeeding at four months postpartum, giving fluids on the first day after birth, bottle feeding	Exclusive breastfeeding rates were significantly higher, 79% in the intervention group and 48% in the control group at 3 months (OR 4.0, 95% CI 3.0–5.4). The mean duration of exclusive breastfeeding in the intervention group was 122 days, versus 41 days in the control group	Prevalence of Exclusive breastfeeding was significantly higher at 5 months, (70%) for the intervention group and (6%) for the control group. Difference = 64%; 95% CI 57%-71%). 64% of the intervention group initiated breastfeeding in the first hour compared to 15% in the control group	At 6 months postpartum 59.6% in the intervention group and 28.6% in the control group exclusively breastfed. (Adj. HR = 0.4, 95% CI: 0.3–0.6). Prelacteal feeding was less likely among intervention mothers (Adj. RR = 0.5, 95% CI 0.3–0.8)	In Uganda, exclusive breastfeeding prevalence at 12 weeks was 82% (intervention) vs. 44% (control) (PR 1.89, 95% CI 1.70–2.11), and at 24 weeks, 59% vs. 15% (PR 3.83, 95% CI 2.97–4.95). In Burkina Faso, at 12 weeks, it was 79% vs. 35% (PR 2.29, 95% CI 1.33–3.92), and at 24 weeks, 73% vs. 22% (PR 3.33, 95% CI 1.74–6.38). In South Africa, at 12 weeks, it was 10% vs. 6% (PR 1.72, 95% CI 1.12–2.63), and at 24 weeks, 2% vs. < 1% (PR 5.70, 95% CI 1.33–24.26)	The prevalence of exclusive breastfeeding at 6 months was 23.6% in the home-based intervention group, 9.2% in the health facility-based intervention group and 5.6% in the control group. In the home-based intervention group mothers had four times increased likelihood of exclusive breastfeeding compared with controls (Adj. RR = 4·01, 95% CI: 2.30–7·01). No significant difference was found comparing exclusive breastfeeding in health- facility based intervention group and control group

*OR* refers to odds ratio, *HR* refers to hazard ratio, *RR* refers to risk ratio

^a^Articles highlighted in this table are those which are included in the discussion of this manuscript. For a comprehensive overview of all breastfeeding interventions in LMICs overall, please see Pezley et al. 2019 [30]

In our study women with mild anxiety levels who received the intervention had over two-fold higher odds of reporting recent breastfeeding at the six-week postpartum time point compared to controls. This association was not significant among intervention participants who had moderate to high levels of anxiety, indicating heightened efficacy of HMHB in the low anxiety group. Numerous CBT interventions have produced significant results in improving mental health of the individuals with anxiety, yet they have not specified effects based on the severity of anxiety [9, 10, 15, 36, 37, 38]. To affect breastfeeding outcomes for women with severe mental health problems, a more intense intervention may be needed, whereas this CBT-based psychosocial intervention potentially fostered breastfeeding by stimulating the responsiveness of mothers with mild anxiety.

The HMHB intervention, while effective in promoting early breastfeeding initiation and continuation among those completing the program, primarily concentrated on addressing perinatal anxiety. It only briefly touched upon breastfeeding promotion in the final session. Therefore, given an association between perinatal common mental health disorders and breastfeeding, the positive effects we observed may also have been due to the ability of the program to reduce anxiety and depression. This is supported by the literature. For example in a study conducted in Turkey by Çiftçi and Arikan in 2012, an association was observed between the presence of postnatal maternal anxiety levels and a decline in the exclusivity and continuation of breastfeeding [39]. Research recommends actively monitoring and appropriately managing maternal anxiety during the postpartum period to foster optimal breastfeeding practices [39, 40, 41]. It has been suggested that use of intrapartum analgesia (fentanyl) during labor and antidepressants used during pregnancy (including selective serotonin reuptake inhibitors (SSRIs) and serotonin-norepinephrine reuptake inhibitors (SNRIs)) significantly reduce breastfeeding [40, 41], which highlights the need for comprehensive lactation support in maternal healthcare especially for women with mental health conditions. Another single-session intervention (coupled with monthly telephone support), targeted postpartum mothers with depression while providing information on mental health, the benefits of breastfeeding and tips for successful breastfeeding. The findings indicated significant improvements in exclusive breastfeeding and breastfeeding practices, which were correlated with a reduction in postpartum depression within three months [42].

The prevailing social norms regarding breastfeeding in Pakistani culture discourage breastfeeding by not providing adequate support to mothers, making them feel uncomfortable breastfeeding in public and at the workplace [43]. Our study suggests that a CBT intervention for promoting breastfeeding practices among anxious women holds promise in Pakistan and potentially in other similar LMIC settings. The results highlight the importance of the full dose of the intervention in supporting successful breastfeeding for anxious mothers in a setting that falls short of WHO breastfeeding targets. In light of these findings, further investigation through mediation analyses could offer valuable insights into the mechanisms explaining the association between the full dose of the intervention and breastfeeding.

### Strengths and limitations

One notable strength of our study is that we used a randomized controlled trial design to evaluate the effects of an the intervention for women with at least mild prenatal anxiety symptoms, a high risk group for discontinued breastfeeding [37]. However, given this focus, our findings may not be generalizable to non-anxious women of reproductive age in Pakistan. Regarding recruitment, of the 1307 women who were eligible, 107 deeclined to participate resulting in a mismatch between the eligible population and those who participated. However, we lacked information on those who did not participate in order to evaluate whether volunteer bias was a problem in our study (i.e., if those who declined to participate differed from those who agreed to participate). The trial was not originally designed to evaluate breastfeeding as a primary outcome, which may have led to the study being underpowered to assess breastfeeding. Further, since our study was hospital-based, results may not be transferrable to women in rural areas or those who typically give birth at home. Another limitation pertains to our reliance on retrospective recall of breastfeeding that corresponded to the initial 24-h period following birth, which was asked at six weeks postpartum. Further, the use of only one timepoint to assess breastfeeding may have also resulted in our missing important variation and changes in breastfeeding behaviors over time. The omission of some relevant variables, such as delivery type (e.g. vaginal versus caesarean), mother-newborn separation, prior breastfeeding experience, intention to breastfeed, and medication usage during labor makes it difficult to attribute our findings solely to the intervention, underscoring the need for additional research. Finally, we had a high rate of loss to follow-up, the analysis of which showed differences between participants enrolled and who completed the study related to gestational age at birth, education level, and income [16]. This may be partially due to the fact our data collection overlapped with the COVID-19 pandemic, during which many women were afraid to use hospital facilities [44].

## Conclusion

The study did not reveal any significant effect of the anxiety focused psychosocial intervention on breastfeeding outcomes in intent-to-treat analyses. However, findings of more robust effects in women with mild anxiety (compared to more severe cases) support the potential use of HMHB in promoting recent breastfeeding practices specifically among women who have mild symptoms of anxiety. A different kind or more intense intervention may be needed to promote breastfeeding among women with higher levels of anxiety. Moreover, several other factors contribute to the challenges of breastfeeding including family support, employment, and childcare, affecting the overall lower breastfeeding rates in Pakistan [43]. Future studies are needed to investigate the intersecting role of these factors in the promotion of breastfeeding, and to understand why we observed stronger effects for women with only mild anxiety in our study. More research is needed to establish if HMHB is effective for the general population of non-anxious women and how it could be tailored to be effective for the most anxious women.

### Supplementary Information

Supplementary Material 1.

## Data Availability

The data used in this study can be accessed at the US National Institutes of Health, National Institute of Mental Health (NIMH) Data Archive: https://nda.nih.gov/.

## Abbreviations

CBT: Cognitive behavioural therapy
CI: Confidence interval
HADS: Hospital Anxiety Depression Scale
HFH: Holy Family Hospital
HMHB: Happy Mother - Healthy Baby
ITT: Intent-to-treat
LMIC: Lower- and middle-income countries
MDE: Major depressive episode
MSPSS: Multidimensional Scale of Social Support
OR: Odds ratio
SCID: Structured Clinical Interview for DSM V Diagnoses
SD: Standard deviation
WHO: World Health Organization
