# Supplementary material for: Impact of an intervention for perinatal anxiety on breastfeeding: findings from the Happy Mother—Healthy Baby randomized controlled trial in Pakistan
Source: Int Breastfeed J. 2024 Aug 2;19:53. doi: 10.1186/s13006-024-00655-8 (PMC11295719; doi:10.1186/s13006-024-00655-8)
Supplement: Supplementary file 1 — Supplementary Material 1. [file 13006_2024_655_MOESM1_ESM.docx]

**Supplemental Table 1:** Breastfeeding in relation to postpartum depression and anxiety levels measured with the Hospital Anxiety and Depression Scale (HADS)

| **Breastfeeding type** | **Estimate*** | **95% CI** |
| --- | --- | --- |
| ***Association with postpartum depression level (HADS depression)*** | | |
| Early breastfeeding | -1.17 | (-2.06, -0.29) |
| Exclusive early breastfeeding | -0.68 | (-1.94, 0.58) |
| Recent breastfeeding | -0.45 | (-1.25, 0.35) |
| Exclusive recent breastfeeding | -0.72 | (-1.96, 0.53) |
| ***Association with postpartum anxiety level (HADS anxiety)*** | | |
| Early breastfeeding | -0.61 | (-1.37, 0.16) |
| Exclusive early breastfeeding | -0.75 | (-1.83, 0.34) |
| Recent breastfeeding | 0.10 | (-0.58, 0.79) |
| Exclusive recent breastfeeding | -0.35 | (-1.42, 0.72) |

**Supplemental Table 2.** Description of 298 women with mild anxiety at enrolment (HADS anxiety < 11) with measured breastfeeding at six-weeks postpartum enrolled in the HMHB trial to be used in the intent-to-treat analysis

|  | **Overall**  **(N=298)** | **Intervention Arm**  **(N=149)** | **Control Arm**  **(N=149)** |
| --- | --- | --- | --- |
|  | Mean (SD) | Mean (SD) | Mean (SD) |
| Age (years) | 24.9 (4.6) | 24.8 (4.4) | 25.0 (4.8) |
| Gestational Age (weeks) | 38.1 (2.1) | 38.1 (2.2) | 38.1 (2.0) |
|  |  |  |  |
| Stress at enrolment (PSS-10) | 19.9 (2.4) | 19.9 (2.5) | 19.9 (2.3) |
|  |  |  |  |
| Anxiety at enrolment (HADS) | 9.4 (0.7) | 9.3 (0.7) | 9.5 (0.7) |
| Depression at enrolment (HADS) | 5.7 (2.0) | 5.7 (2.2) | 5.7 (1.9) |
| Major social support (MSPSS) | 3.3 (0.8) | 3.2 (0.8) | 3.3 (0.8) |
| Social support from family (MSPSS) | 3.2 (0.9) | 3.2 (0.9) | 3.2 (0.9) |
| Social support from friend (MSPSS) | 3.2 (0.9) | 3.2 (0.9) | 3.2 (0.9) |
| Maternal age ≤ 25 | 154 (52%) | 76 (51%) | 78 (52%) |
| Child’s gender (Male) | 144 (48%) | 69 (46%) | 75 (50%) |
| First pregnancy (Yes) | 89 (30%) | 43 (29%) | 46 (31%) |
| ≥ 1 child from a prior pregnancy | 168 (56%) | 82 (55%) | 86 (58%) |
| History of stillbirth or miscarriage (Yes) | 125 (42%) | 66 (44%) | 59 (40%) |
|  |  |  |  |
| Education level |  |  |  |
| ≤ Primary school | 66 (22%) | 34 (23%) | 32 (21%) |
| Middle school – matriculation | 140 (47%) | 72 (48%) | 68 (46%) |
| ≥ Intermediate | 92 (31%) | 43 (29%) | 49 (33%) |
| Family structure |  |  |  |
| Nuclear | 93 (31%) | 46 (31%) | 47 (32%) |
| Joint (parents) | 104 (35%) | 52 (35%) | 52 (35%) |
| Extended (parents and siblings) | 101 (34%) | 51 (34%) | 50 (34%) |
| Monthly income (PKR ($) |  |  |  |
| Low (<20,000 (<$100) | 126 (42%) | 59 (40%) | 67 (45%) |
| Middle (20,000 – 35000 ($100-$1256) | 118 (40%) | 64 (43%) | 54 (36%) |
| High (>35,000 (>$125) | 54 (18%) | 26 (17%) | 28 (19%) |
|  |  |  |  |
| Anxiety at enrolment (HADS) |  |  |  |
| Mild (≥ 8 to ≤10) | 298 (100%) | 149 (100%) | 149 (100%) |
| Moderate (≥ 11 to ≤15) | 0 (0%) | 0 (0%) | 0 (0%) |
| Severe (≥ 16 to ≤ 21) | 0 (0%) | 0 (0%) | 0 (0%) |

Note: Social support is considered the provision of emotional, informational, appraisal, and instrumental from others in one’s social network. For family structure, nuclear family structure refers to the participant, her husband and children; joint family structure refers to living with her husband, children and in-laws; extended family structure includes living with not only in-law parents but also sister- and brothers-in-laws and potentially their families.

**Supplemental Table 3.** Description of 422 women with moderate to severe anxiety at enrolment (HADS anxiety 11 - 21) with measured breastfeeding at six-weeks postpartum enrolled in the HMHB trial to be used in the intent-to-treat analysis

|  | **Overall**  **(N=422)** | **Intervention Arm**  **(N=213)** | **Control Arm**  **(N=209)** |
| --- | --- | --- | --- |
|  | Mean (SD) | Mean (SD) | Mean (SD) |
| Age (years) | 25.4 (4.6) | 25.3 (4.9) | 25.6 (4.3) |
| Gestational Age (weeks) | 38.1 (2.0) | 38.1 (2.1) | 38.2 (1.9) |
|  |  |  |  |
| Stress at enrolment (PSS-10) | 19.9 (2.6) | 20.0 (2.7) | 19.8 (2.5) |
|  |  |  |  |
| Anxiety at enrolment (HADS) | 12.4 (1.5) | 12.5 (1.5) | 12.4 (1.5) |
| Depression at enrolment (HADS) | 7.5 (3.0) | 7.7 (3.1) | 7.2 (2.8) |
| Major social support (MSPSS) | 3.2 (0.9) | 3.2 (1.0) | 3.3 (0.9) |
| Social support from family (MSPSS) | 3.2 (1.0) | 3.1 (1.0) | 3.3 (1.0) |
| Social support from friend (MSPSS) | 3.2 (1.0) | 3.2 (1.0) | 3.2 (0.9) |
| Maternal age ≤ 25 | 183 (43%) | 96 (45%) | 87 (42%) |
| Child’s gender (Male) | 208 (49%) | 109 (51%) | 99 (47%) |
| First pregnancy (Yes) | 118 (28%) | 55 (26%) | 63 (30%) |
| ≥ 1 child from a prior pregnancy | 243 (58%) | 122 (57%) | 121 (58%) |
| History of stillbirth or miscarriage (Yes) | 178 (42%) | 94 (44%) | 84 (40%) |
|  |  |  |  |
| Education level |  |  |  |
| ≤ Primary school | 111 (26%) | 58 (27%) | 53 (25%) |
| Middle school – matriculation | 196 (46%) | 95 (45%) | 101 (48%) |
| ≥ Intermediate | 115 (27%) | 60 (28%) | 55 (26%) |
| Family structure |  |  |  |
| Nuclear | 138 (33%) | 73 (34%) | 65 (31%) |
| Joint (parents) | 144 (34%) | 69 (32%) | 75 (36%) |
| Extended (parents and siblings) | 140 (33%) | 71 (33%) | 69 (33%) |
| Monthly income (PKR ($) |  |  |  |
| Low (<20,000 (<$100) | 192 (45%) | 97 (46%) | 95 (45%) |
| Middle (20,000 – 35000 ($100-$1256) | 167 (40%) | 79 (37%) | 88 (42%) |
| High (>35,000 (>$125) | 63 (15%) | 37 (17%) | 26 (12%) |
|  |  |  |  |
| Anxiety at enrolment (HADS) |  |  |  |
| Mild (≥ 8 to ≤10) | 0 (0%) | 0 (0%) | 0 (0%) |
| Moderate (≥ 11 to ≤15) | 403 (95%) | 205 (96%) | 198 (95%) |
| Severe (≥ 16 to ≤ 21) | 19 (5%) | 8 (4%) | 11 (5%) |

Note: Social support is considered the provision of emotional, informational, appraisal, and instrumental from others in one’s social network. For family structure, nuclear family structure refers to the participant, her husband and children; joint family structure refers to living with her husband, children and in-laws; extended family structure includes living with not only in-law parents but also sister- and brothers-in-laws and potentially their families.

**Supplemental Table 4.** Description of 358 women in the control arm, and 195 women in the intervention arm receiving six intervention sessions (“intervention completers”) in the HMHB trial with measured breastfeeding at six-weeks postpartum enrolled.

|  | **Overall**  **(N=553)** | **Intervention Arm**  **(N=195)** | **Control Arm**  **(N=358)** |
| --- | --- | --- | --- |
|  | Mean (SD) | Mean (SD) | Mean (SD) |
| Age (years) | 25.3 (4.6) | 25.3 (4.8) | 25.3 (4.5) |
| Gestational Age (weeks) | 38.2 (2.0) | 38.3 (2.0) | 38.1 (1.9) |
|  |  |  |  |
| Stress at enrolment (PSS-10) | 19.9 (2.5) | 20.1 (2.6) | 0.97 (1.30) |
|  |  |  |  |
| Anxiety at enrolment (HADS) | 11.1 (1.9) | 11.1 (2.0) | 11.2 (1.9) |
| Depression at enrolment (HADS) | 6.7 (2.7) | 6.8 (2.9) | 6.6 (2.6) |
| Major social support (MSPSS) | 3.2 (0.9) | 3.2 (0.9) | 3.6 (0.9) |
| Social support from family (MSPSS) | 3.2 (0.9) | 3.1 (1.0) | 3.5 (0.9) |
| Social support from friend (MSPSS) | 3.2 (0.9) | 3.1 (1.0) | 2.7 (1.3) |
| Maternal age ≤ 25 | 261 (47%) | 96 (49%) | 220 (61%) |
| Child’s gender (Male) | 260 (47%) | 86 (44%) | 174 (49%) |
| First pregnancy (Yes) | 157 (28%) | 48 (25%) | 109 (30%) |
| ≥ 1 child from a prior pregnancy | 322 (58%) | 115 (59%) | 207 (58%) |
| History of stillbirth or miscarriage (Yes) | 231 (42%) | 88 (45%) | 143 (40%) |
|  |  |  |  |
| Education level |  |  |  |
| ≤ Primary school | 136 (25%) | 51 (26%) | 85 (24%) |
| Middle school – matriculation | 264 (48%) | 95 (49%) | 169 (47%) |
| ≥ Intermediate | 153 (28%) | 49 (25%) | 104 (29%) |
| Family structure |  |  |  |
| Nuclear | 179 (32%) | 67 (34%) | 112 (31%) |
| Joint (parents) | 187 (34%) | 62 (32%) | 125 (35%) |
| Extended (parents and siblings) | 187 (34%) | 66 (34%) | 107 (30%) |
| Monthly income (PKR ($) |  |  |  |
| Low (<20,000 (<$100) | 244 (44%) | 82 (42%) | 172 (48%) |
| Middle (20,000 – 35000 ($100-$1256) | 224 (41%) | 82 (42%) | 138 (39%) |
| High (>35,000 (>$125) | 85 (15%) | 31 (16%) | 41 (11%) |
|  |  |  |  |
| Anxiety at enrolment (HADS) |  |  |  |
| Mild (≥ 8 to ≤10) | 234 (42%) | 85 (44%) | 149 (42%) |
| Moderate (≥ 11 to ≤15) | 302 (55%) | 104 (53%) | 198 (55%) |
| Severe (≥ 16 to ≤ 21) | 17 (3%) | 6 (3%) | 11 (3%) |

Note: Social support is considered the provision of emotional, informational, appraisal, and instrumental from others in one’s social network. For family structure, nuclear family structure refers to the participant, her husband and children; joint family structure refers to living with her husband, children and in-laws; extended family structure includes living with not only in-law parents but also sister- and brothers-in-laws and potentially their families.

| Table 5: Comparison of psychosocial interventions for breastfeeding in lower- and middle-income countries^†^ | | | | | |
| --- | --- | --- | --- | --- | --- |
|  | **HMHB - Pakistan** | **Syria** (32) | **India** (33) | **Bangladesh** (34) | **Pakistan** (13) |
| Intervention  goal | To use cognitive behavioral therapy to reduce prenatal anxiety and to facilitate participants wellbeing, social support, and bonding with their baby during pregnancy | To provide medical follow up, educate provide emotional support, check on breastfeeding, check on maternal-child relationship, discuss problems and help women who have given birth, discuss family planning | To promote exclusive breastfeeding until 6 months of age (as well as assess effects on diarrhea and child growth) | To educate and counsel mothers about exclusive breastfeeding and early initiation of breastfeeding | To use cognitive-behavioral therapy to increase the rate and duration of exclusive breastfeeding in the first six months postpartum |
| Time point | Antenatal | Postnatal | Postnatal | Antenatal and postnatal | Antenatal and postnatal |
| Frequency of delivery | Weekly for 5 visits in early to mid-pregnancy, with a 6^th^ visit in the 3^rd^ trimester | At least once or in a series of 4 home visits | A series of 12 monthly visits until the child reached the age of one | 15 sessions | 7 sessions |
| Location | Health facility | Home | Home | Home | Home |
| Delivery agent | Non-specialized providers | Trained midwives | Community health workers and nutrition workers | Peer counsellors | Community health workers |
| Outcome(s)  related to breastfeeding | Exclusive and recent breastfeeding both within 24 hours of birth and within 24 hours of an assessment at six weeks postpartum | Exclusive breastfeeding and breastfeeding practices (see below for examples) at 4 months postpartum | Primary: Exclusive breastfeeding at 3 months postpartum; Secondary: Exclusive breastfeeding at 4, 5, and 6 months of life. | Prevalence of exclusive breastfeeding at 5 months postpartum and timing of initiation of breastfeeding | Rate and duration of exclusive breastfeeding at 6 months postpartum |
| Result(s)  related to breastfeeding | HMHB had a marginally significant impact on early breastfeeding i.e. in the first 24 hours of life (75.4% HMHB vs. 69.0% controls; OR=1.4, 95% CI: 0.99-1.92). In unadjusted per protocol analyses, HMHB increased the odds of early (OR=1.7, 95% CI:1.2-2.6) and recent breastfeeding i.e. measured in the prior 24 hours at six-weeks after birth (OR=2.1, 95% CI:1.1-4.0). | A significantly higher proportion of mothers who received four doses or one dose of the intervention, respectively, exclusively breastfed their infants (28.5% and 30%, respectively) compared to controls who received no intervention (20%), p=0.02. However, no differences were found between these groups for other breastfeeding outcomes, e.g. breastfeeding at four months postpartum, giving fluids on the first day after birth, bottle feeding. | Exclusive breastfeeding rates were significantly higher, 79% in the intervention group and 48% in the control group at 3 months (OR=4.0, 95% CI 3.0-5.4). The mean duration of exclusive breastfeeding in the intervention group was 122 days, versus 41 days in the control group. | Prevalence of Exclusive breastfeeding was significantly higher at 5 months, (70%) for the intervention group and (6%) for the control group. Difference=64%; 95% CI 57%-71%). 64% of the intervention group initiated breastfeeding in the first hour compared to 15% in the control group. | At 6 months postpartum 59.6% in the intervention group and 28.6% in the control group exclusively breastfed. (Adj. HR= 0.4, 95% CI: 0.3–0.6). Prelacteal feeding was less likely among intervention mothers (Adj. RR=0.5, 95% CI 0.3-0.8) |

|  | **Sessions received** | **N obs** | **N breastfeeding (%)** | **p*** |
| --- | --- | --- | --- | --- |
| Early breastfeeding | No sessions | 358 | 247 (69%) | 0.090 |
|  | Intervention arm without booster sessions | 286 | 216 (76%) |  |
|  | Intervention arm with booster sessions | 76 | 57 (75%) |  |
| Early breastfeeding | No sessions | 358 | 34 (9%) | 0.125 |
|  | Intervention arm without booster sessions | 286 | 36 (13%) |  |
|  | Intervention arm with booster sessions | 76 | 11 (14%) |  |
| Recent breastfeeding | No sessions | 358 | 309 (86%) | 0.219 |
|  | Intervention arm without booster sessions | 286 | 260 (91%) |  |
|  | Intervention arm with booster sessions | 76 | 67 (88%) |  |
| Exclusive recent breastfeeding | No sessions | 358 | 175 (49%) | 0.802 |
|  | Intervention arm without booster sessions | 286 | 139 (49%) |  |
|  | Intervention arm with booster sessions | 76 | 39 (51%) |  |
| *Significance determined by Cochrane Armitage test. | |  |  |  |

**Table 6: Dose response analysis among HMHB trial participants with six week postpartum follow-up**
